# A proposed method of grading malaria chemoprevention efficacy

**DOI:** 10.1093/trstmh/trad042

**Published:** 2023-07-10

**Authors:** N J White, C Bonnington, F H Nosten

**Affiliations:** Mahidol-Oxford Tropical Medicine Research Unit, Faculty of Tropical Medicine, Mahidol University, Bangkok 10400, Thailand; Centre for Tropical Medicine and Global Health, Nuffield Department of Medicine, University of Oxford, OX3 7LJ, UK; Centre for Tropical Medicine and Global Health, Nuffield Department of Medicine, University of Oxford, OX3 7LJ, UK; Malaria Consortium, London, UK; Centre for Tropical Medicine and Global Health, Nuffield Department of Medicine, University of Oxford, OX3 7LJ, UK; Shoklo Malaria Research Unit, Mahidol‐Oxford Tropical Medicine Research Unit, Faculty of Tropical Medicine, Mahidol University, Bangkok 10400, Thailand

## Abstract

The efficacy and effectiveness of antimalarial drugs are threatened by increasing levels of resistance and therefore require continuous monitoring. Chemoprevention is increasingly deployed as a malaria control measure, but there are no generally accepted methods of assessment. We propose a simple method of grading the parasitological response to chemoprevention (focusing on seasonal malaria chemoprevention) that is based on pharmacometric assessment.

## Introduction

Seasonal malaria chemoprevention (SMC) is the administration of treatment doses of antimalarial drugs to children at monthly intervals to suppress malaria infections in areas of highly seasonal transmission. It is therefore a form of antimalarial chemoprophylaxis. For the most part, SMC (formerly known as intermittent preventive treatment in children [IPTc]) has used a combination of three drugs, sulphadoxine–pyrimethamine with amodiaquine (SPAQ), in children 3–59 months of age. SMC has been deployed across the Sahel region of Africa, a belt of intense seasonal malaria transmission. SPAQ is given monthly for 3–5 months [1]. The excellent results of large prospective evaluations, which overall have shown an approximate 75% reduction in clinical malaria, resulted in adoption of SMC as policy by the World Health Organization (WHO) in 2012 [2, 3]. The new policy was accompanied by a recommendation that methods be developed to assess and monitor the continued effectiveness of SMC: ‘Drug resistance monitoring and system evaluation should be supported or instituted, including systems to assess the number of breakthrough infections and their intervals from the last dose of SMC’ [3]. But this recommendation was not followed and today, >10 y later and after hundreds of millions of doses of SPAQ have been deployed, there is still no accepted or validated method for assessing this increasingly widely deployed intervention. The WHO also recommends other forms of chemoprevention in African children, notably intermittent preventive treatment in infants (IPTi), i.e. giving treatment doses of SP delivered together with the Expanded Program on Immunization vaccines at 2, 3 and 9 months of age. This intervention has not been widely adopted. There is also no accepted or validated method of assessing IPTi or any of the currently recommended forms of preventive antimalarial chemotherapy.

Meanwhile, SMC deployment has been scaled up and now has spread across Africa. In 2022 the WHO broadened substantially their original recommendations for SMC (which were confined to the Sahel) to other geographic regions in Africa (where drug resistance is worse) and removed restrictions on the number of monthly cycles or age [4, 5]. Similarly, the recommendations for IPTi were loosened to include a broader age range and timing of drug administration. IPTi was renamed perennial malaria chemoprevention (PMC). The effectiveness of PMC depends on a drug that is failing in many malaria-endemic regions. Resistance to sulphadoxine and pyrimethamine are widespread. But an earlier restriction for IPTi, based on molecular markers of resistance indicative of high levels of resistance, was removed [4] and no method to assess and monitor the continued effectiveness of this new PMC approach was investigated.

Funding to support malaria control is not unlimited. Clearly it is important for national control programs, agencies and donors to know that the preventive interventions they are providing do work and that they are not supporting interventions that are ineffective. Continued effective suppression of malaria by SMC justifies the considerable expense and effort. But if the current chemoprevention interventions start to lose their effectiveness, this should be recognized quickly and alternative approaches should be deployed. Historically, the use of failing or useless antimalarial drugs has continued for many years and has resulted in substantial human and financial costs [6]. This should not be allowed to happen again. All malaria interventions, and particularly those vulnerable to resistance, need robust and operationally feasible methods of assessment so that their effectiveness can be monitored.

## Chemoprevention pharmacometrics

SMC differs fundamentally from the treatment of clinical malaria. In SMC, treatment doses of antimalarials are given to healthy children. Many of these children are infected with malaria parasites, but at densities in the blood that do not make them ill. In most cases they have already controlled their malaria infections. As a result, when they take an antimalarial drug, the therapeutic response is substantially better than in a symptomatic infection, which, by definition, has not been controlled by the host and is usually associated with parasite numbers that are orders of magnitude higher [7, 8]. Nevertheless, the drugs still have to provide some suppressive benefit during their long elimination phase in order to prevent new infections from becoming patent (and potentially transmitting) until the next monthly treatment dose is given [9].

Pharmacometric antimalarial resistance monitoring (PARM) describes a simple method of assessing chemopreventive antimalarial drug efficacy [8]. It relies primarily on dried filter paper samples from which measurements of both drug levels and quantitative polymerase chain reaction (qPCR) parasite densities are made [8]. PARM does not require sample refrigeration or weekly follow-up. The key measure is the qPCR parasite density estimate at 28 d (i.e. just before the next round of SMC). Filter paper qPCR on blood spots has a density limit of detection of around 1–5 parasites/µl [10]. In a day 28 malaria-positive sample, drug measurement allows the critical distinction between low drug exposure and drug resistance to be made. Parasite DNA in the same filter paper samples can be used for evaluation of molecular markers of drug resistance and distinction of reinfection from recrudescence. Taking blood slides at day 28 to identify gametocytaemia is also informative if breakthrough rates are high (≥M2, see below). A filter paper drug assay on day 7 provides additional information and is particularly valuable for estimating exposure to more rapidly eliminated antimalarial drugs [8].

**Figure 1.**
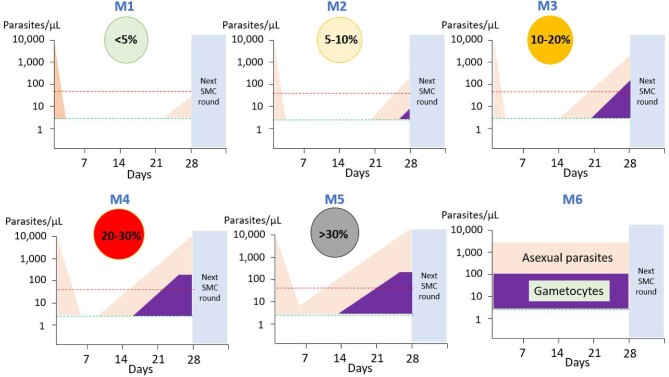
The proposed Mahidol Oxford Tropical Medicine Research Unit (MORU) grading of malaria chemoprevention effectiveness has six levels ranging from fully effective (M1) to completely ineffective (M6). Asexual malaria parasites, if present, should be cleared by the SMC dose and reinfections suppressed for at least 28 days. The proportion of subjects who test qPCR positive for *Plasmodium falciparum* at day 28 in filter paper samples provides the primary effectiveness measure. At higher levels of SMC failure (≥M3), gametocytaemia is detectable by thick blood film microscopy and the infection is therefore likely transmissible. Initially all breakthrough infections are newly acquired but with very high levels of failure (≥M5), some infections detected at day 28 are recrudescences.

We propose a simple grading system to interpret the results of PARM and thus assess SMC effectiveness (Figure 1). This system should be modified and refined as monitoring results are obtained in different areas and with different drugs.

## Proposed grading system for malaria chemoprevention efficacy

The proposed grading depends primarily on the prevalence of filter paper–based qPCR-estimated parasitaemia 28 d after starting SMC. This does not discriminate between asexual and sexual parasitaemia. This value is interpreted in relation to simultaneously measured drug levels. Finding recurrent parasitaemia in the presence of expected drug concentrations suggests resistance. Detection of gametocytaemia by microscopy informs the estimation of drug resistance selection pressures and transmissibility, but it is not used in the proposed grading.

### M1

Even with fully sensitive malaria parasites and highly effective antimalarial medicines there are always some subjects who have low drug exposures (for various reasons including poor adherence, vomiting, pharmacokinetic variability and low drug quality) [9]. An effective SMC or PMC should have <5% of recipients with a filter paper qPCR-detectable malaria parasite density at 28 d.

### M2

As chemoprevention begins to fail (either because of drug resistance or low drug exposures), the proportion of day 28 detectable infections increases to 5–10%. Gametocytaemia is present in a few subjects but gametocyte densities are below the level of microscopy detection and unlikely to transmit. Suppression of further asexual-stage multiplication by the next chemoprevention dose prevents generation of transmissible gametocyte densities.

### M3

The proportion of day 28 detectable infections is 10–20% and some subjects do have microscopy-detectable gametocytaemia at day 28, which is presumably transmissible. This is evidence of the selective pressure on drug resistance. Nearly all recurrent infections are new infections.

### M4

The proportion of day 28 detectable infections is 20–30% and many of these breakthrough infections now have microscopy-detectable gametocytaemia. Some of the breakthrough infections may be associated with illness. The majority of recurrent infections are still new infections.

### M5

The proportion of day 28 detectable infections now exceeds 30% and many of these infections have microscopy-detectable gametocytaemia. Some recurrences are recrudescences, but most recurrent infections are new infections and many are symptomatic.

### M6

Chemoprevention is completely ineffective. The proportion of day 28 subjects who are parasitaemic is similar to that at baseline and there is little or no reduction in parasite prevalence or densities during the 28 d.

## Prevention of clinical illness

This proposed approach to the assessment of malaria chemoprevention, based on basic pharmacometric principles [8], is designed to assess effectiveness and inform policies. In antimalarial treatment therapeutic efficacy assessments, malaria is detected actively and prevention of recurrent clinical illness is a secondary endpoint [11]. For chemoprevention, although effectiveness in preventing parasitaemia, reducing resistance selection, preventing transmission of drug-exposed infections and preventing clinical malaria are all linked, it is prevention of illness that is most important. This is the primary justification for deploying antimalarial chemoprevention. It is assumed that parasitological and clinical efficacy are closely linked, although in high-transmission settings the majority of infections in older children are asymptomatic. Rising rates of parasitological failure are a harbinger of clinical failure. The grading system proposed would warrant concern at M2 and particularly at M3 and consideration of policy change at M3 and above. The proportion of breakthrough infections that are symptomatic should be noted. Body temperatures should be measured and a brief symptom enquiry made at the time of day 28 screening. In high transmission settings the risk of symptomatic malaria is strongly (inversely) proportional to age. If a significant proportion of breakthrough infections are symptomatic, that would be additional cause for concern, prompting a policy change. Whether the proportion of breakthrough infections that are symptomatic should be included in the grading system will require further study and evaluation.

## Discussion

SMC has been an excellent malaria control intervention across the Sahel and has provided substantial benefit in reducing childhood malaria [12–16]. SPAQ is considered to retain good activity in this region and the intense seasonality of malaria makes the SMC approach operationally feasible and thus cost effective. In contrast, in East and Central Africa, where transmission is intense, malaria is often less seasonal and drug resistance is much worse, the benefit of unfettered deployment of SPAQ malaria chemoprevention is uncertain. In a responsible widescale deployment of an antimalarial (or indeed any anti-infective), intervention effectiveness must be measured. The method of assessment described here is robust but simple and operationally feasible, and it provides a quantifiable measure of effectiveness (encompassing efficacy and operational performance). It also informs on the cause of the chemoprevention failure. This grading is an unvalidated proposal that will need refinement and improvement as information accrues. There are obviously risks associated with the newly expanded WHO chemoprevention policies and their implementation without effectiveness monitoring in areas with high levels of drug resistance, so information on chemoprevention effectiveness should be gathered now to guide responsible deployment.

SMC and PMC should only be deployed with drugs that are effective in areas where there is high transmission and a high burden of illness. In this context, a substantial proportion of the target population is parasitaemic (by qPCR) at the beginning of the malaria transmission season. Obviously, if only a minority of subjects are parasitaemic before SMC is deployed and transmission is relatively low or the study is done at the wrong time in relation to the transmission season, then there will be few breakthrough infections at day 28. It could thus be concluded incorrectly that SMC was effective. Measuring the baseline prevalence addresses this question, although in some areas with intensely seasonal transmission the first round of SMC might be given just before the rapid increase in malaria prevalence.

In an attempt to justify continued use of the failing antimalarial drug chloroquine, it was once argued that control of parasitaemia was sufficient to justify antimalarial drug deployment and it was suggested that clinical benefits could be dissociated from parasitological responses in malaria [17]. This is generally wrong. That costly mistake should not be repeated. There are other effective drugs that can be used for SMC. If SPAQ chemoprevention is found to be effective, it will reassure control programs and donors that their substantial efforts and investments are being rewarded. If it is found to be failing, then it must be substituted. Continued deployment of failing drugs will result in increased transmission and the associated morbidity and mortality, and it will accelerate resistance. Continued measurement of chemoprevention effectiveness aims to prevent this from happening.

## Data Availability

Not applicable.
